# Human endometrial regenerative cells alleviate carbon tetrachloride-induced acute liver injury in mice

**DOI:** 10.1186/s12967-016-1051-1

**Published:** 2016-10-22

**Authors:** Shanzheng Lu, Ganggang Shi, Xiaoxi Xu, Grace Wang, Xu Lan, Peng Sun, Xiang Li, Baoren Zhang, Xiangying Gu, Thomas E. Ichim, Hao Wang

**Affiliations:** 1 Department of General Surgery, Tianjin Medical University General Hospital, 154 Anshan Road, Heping District, Tianjin, 300052 China; 2 Tianjin General Surgery Institute, Tianjin, China; 3 Faculty of Medicine, University of Toronto, Toronto, ON Canada; 4 Department of General Surgery, Affiliated Hospital of Weifang Medical University, Shandong, China; 5 Department of Gynecology and Obstetrics, Tianjin Medical University General Hospital, Tianjin, China; 6 Immune Advisors LLC, San Diego, CA 92121 USA

**Keywords:** Endometrial regenerative cells, Acute liver injury, Anti-inflammatory, Immunoregulation

## Abstract

**Background:**

The endometrial regenerative cell (ERC) is a novel type of adult mesenchymal stem cell isolated from menstrual blood. Previous studies demonstrated that ERCs possess unique immunoregulatory properties in vitro and in vivo, as well as the ability to differentiate into functional hepatocyte-like cells. For these reasons, the present study was undertaken to explore the effects of ERCs on carbon tetrachloride (CCl_4_)–induced acute liver injury (ALI).

**Methods:**

An ALI model in C57BL/6 mice was induced by administration of intraperitoneal injection of CCl_4_. Transplanted ERCs were intravenously injected (1 million/mouse) into mice 30 min after ALI induction. Liver function, pathological and immunohistological changes, cell tracking, immune cell populations and cytokine profiles were assessed 24 h after the CCl_4_ induction.

**Results:**

ERC treatment effectively decreased the CCl_4_-induced elevation of serum alanine aminotransferase (ALT) and aspartate aminotransferase (AST) activities and improved hepatic histopathological abnormalities compared to the untreated ALI group. Immunohistochemical staining showed that over-expression of lymphocyte antigen 6 complex, locus G (Ly6G) was markedly inhibited, whereas expression of proliferating cell nuclear antigen (PCNA) was increased after ERC treatment. Furthermore, the frequency of CD4^+^ and CD8^+^ T cell populations in the spleen was significantly down-regulated, while the percentage of splenic CD4^+^CD25^+^FOXP3^+^ regulatory T cells (Tregs) was obviously up-regulated after ERC treatment. Moreover, splenic dendritic cells in ERC-treated mice exhibited dramatically decreased MHC-II expression. Cell tracking studies showed that transplanted PKH26-labeled ERCs engrafted to lung, spleen and injured liver. Compared to untreated controls, mice treated with ERCs had lower levels of IL-1β, IL-6, and TNF-α but higher level of IL-10 in both serum and liver.

**Conclusions:**

Human ERCs protect the liver from acute injury in mice through hepatocyte proliferation promotion, as well as through anti-inflammatory and immunoregulatory effects.

## Background

Despite unprecedented advances made in modern medicine, acute liver diseases remain a healthcare burden. They can arise from viral infections, autoimmune disorders, ischemia, and xenobiotics such as alcohol, drugs and toxins, and can lead to severe clinical outcomes including hepatorenal syndrome, hepatic encephalopathy, severe infection, multiple organ failure, and even death [1]. To date, orthotropic liver transplantation is the most effective therapeutic option for patients suffering from severe irreversible and life-threatening liver damage; however, the limited availability of donor organs, high costs, and lifelong immunosuppressive therapy has severely restricted its clinical application [2]. Hence, alternative strategies for the treatment of decompensated liver diseases are required.

Recent development in stem cell-based therapeutic strategies have already garnered extensive attention and been introduced to regenerative medicine for hepatic diseases [3–5]. It has been demonstrated that infused mesenchymal stem cells (MSCs) engrafting in the liver facilitate the recovery from chemical-induced acute liver damage [6]. Moreover, MSCs possess the characteristics of immunomodulatory, anti-inflammatory and hypoimmunogenicity, and the potential of differentiating into hepatocyte-like cells. Also, MSCs can promote tissue repair by means of suppressing the local immune reaction, attenuating fibrosis and apoptosis, enhancing angiogenesis and stimulating mitosis and differentiation of tissue-intrinsic reparative cells and stem cells [7, 8]. Currently, bone marrow mesenchymal stem cells (BM-MSCs) have become the focal point for cell therapy in liver regeneration [9, 10]. However, BM-MSCs have low yield, invasive operation and decreased cell numbers that are dependent on donor age [11]. Consequently, it is imperative to identify alternative sources of stem cells with better safety and efficacy profiles.

In 2007, Meng et al. discovered a novel type of adult stem cells derived from human menstrual blood, named endometrial regenerative cells (ERCs). These cells possess a self-renewing, highly proliferative potential as well as a differentiation capacity towards diverse cell lineages in appropriate induction media, thereby overcoming the shortcomings of other conventional stem cell sources and the fear of karyotypic abnormalities during culture [12]. Furthermore, ERCs have proven to be an excellent cell source in the treatment of several experimental disease models, such as critical limb ischemia [13], ulcerative colitis [14], burn injury [15], renal ischemia reperfusion injury [16] and other dysfunctional diseases [17–19]. Moreover, it has been verified that these human cells were not rejected in a xenogeneic animal model [13]. ERCs are more readily available and non-invasive than other adult stem cells, making them a promising donor source for stem cell therapy. Recently, ERCs were found to be capable of differentiating into functional hepatocyte-like cells in vitro [20]. However, whether ERCs could simultaneously suppress inflammatory and immune responses and repair tissue damage following ALI remain obscure. Thus, the aim of this study was to explore the potential role of ERCs in alleviation of carbon tetrachloride (CCl_4_)-induced ALI.

## Methods

### Isolation and Culture of ERCs

ERCs were collected from the menstrual blood of healthy female volunteer donors (20–40 years old) using a urine cup after menstrual blood flow initiated. As previously described [12], mononuclear cells were obtained by standard Ficoll method. ERCs were then expanded from the purified mononuclear cells, which were allowed to attach in the endometrial stem cell culture medium (S-Evans Biosciences, China) overnight at 37 °C in 5 % CO_2_. Non-adherent cells were removed by washing with phosphate-buffered saline (PBS), while adherent cells were cultured until they reached 80–90 % confluence. Cells were trypsinized, sub-cultured and used for experiments during passages 4–7.

### Animals

Healthy male C57BL/6 mice (Aoyide Co., Tianjin,China) weighing 18–20 g and aged 6–8 weeks were housed under conventional experimental environment with 12-h light–dark cycle in the Animal Care Facility, Tianjin General Surgery Institute. The mice had free access to commercial standard mouse diet and water until the time of the study. All experiments were conducted in accordance with the protocols following the Animal Care and Use Committee of Tianjin Medical University (China) according to the Chinese Council on Animal Care guidelines.

### Experimental groups

The preparation of animal model was done as previously described [21, 22]. In brief, 18 mice were randomly assigned to the following three groups (n = 6). (1) Normal control group: mice first receiving intraperitoneal (i.p.) injection of corn oil were then injected with 200 μl PBS intravenously 30 min later. (2) Untreated group: mice first receiving i.p. injection of a single dose of CCl_4_ (Sigma-aldrich, St Louis, United States) for induction of acute liver injury were injected 200 μl PBS intravenously 30 min later. (3) ERC-treated group: mice first receiving i.p. injection of CCl_4_ were injected intravenously with 1 × 10^6^ ERCs at passage 4 resuspended in 200 μl of PBS 30 min later [23]. Mice were sacrificed 24 h after injection of CCl_4_, and blood was collected. Livers and spleens were then promptly removed for analysis or stored frozen at −80 °C.

### Measurement of ALT and AST

Serum alanine aminotransferase (ALT) and aspartate aminotransferase (AST) activities were measured by standard spectrophotometric procedures using a ChemiLab ALT and AST assay kit (IVDLab Co., Ltd., Korea), respectively. Enzyme activities were shown in international unit per liter (IU/L).

### Histological examination

Liver slices were made from part of the left lobes and fixed in 10 % neutral buffered formalin, embedded in paraffin and cut into 5 μm sections. Specimens were dewaxed, hydrated and stained with standard hematoxylin and eosin (H&E) to examine morphology.

### Immunohistochemistry staining

Immunohistochemistry was performed with PCNA and Ly6G antibody as described previously [24]. Briefly, the paraffin specimens were cut into 5 μm, followed by deparaffinization and rehydration. Endogenous peroxides were eliminated with 3 % H_2_O_2_, and antigen retrieval was processed by heating in microwave. Then, the sections were blocked with 5 % bovine serum albumin (BSA) and incubated with anti-mouse PCNA and Ly6G (Abcam, Cambridge, MA) antibodies overnight at 4 °C, respectively. Secondary labeling was achieved by goat anti-rabbit IgG and rabbit anti-rat IgG polyclonal antibody, separately. Horseradish peroxidase-conjugated avidin and brown-colored diaminobenzidine were used to visualize the labeling. Finally, the slides were counterstained with hematoxylin. All of stained sections were photographed using an Olympus inverted microscope (Olympus Imaging America, Center Valley, PA).

### Enzyme-linked immunosorbent assay

The levels of IL-1β, IL-6, TNF-α,and IL-10 in the serum and liver samples taken from mice 24 h after CCl_4_ challenge were measured by ELISA kit (eBiosciences, San Diego, CA, USA) according to the manufacturer’s instructions. ELISA was performed in duplicate for each sample. The preparation of liver homogenate was done as previously described [25]. In short, frozen liver tissues were homogenized in a protein extraction solution (PRO-PREP; Intron biotechnology, Sungnam, Korea), incubated for 30 min on ice and then centrifuged at 13,000 rpm (4 °C) for 10 min.

### Flow cytometry analysis

The phenotype of various immune cells was evaluated by flow cytometry analysis [FACS, Epic XL, Software Expo32 (Beckman coulter)]. Briefly, splenocytes were stained with fluoresent antibodies, including anti-CD3ε-FITC, anti-CD8a-PerCP, anti-CD4-PE/FITC, anti-CD25-PE, anti-Foxp3-PerCP, anti-CD11c-PE, anti-MHCII-FITC (eBiosciences, San Diego, CA, USA), according to the manufacturer’s instructions. The percentage of each phenotype of immune cells was analyzed with the corresponding Flowjo software.

### Labeling of ERCs with PKH26 and in vivo tracking

For in vivo tracking of administered ERCs, cells were isolated and labeled with PKH26 Red Fluorescent Cell Linker Kits (Sigma-aldrich, St Louis, USA), according to the manufacturer’s instructions. Prepared PKH26-labeled ERCs at a final cell concentration of 1 × 10^7^ cells/ml were then injected via tail vein 30 min after ALI induction. Mice were executed 24 h later, and the liver, lung, kidney and spleen were removed and frozen at −20 °C. Fluorescence microscopy was performed to analyze the 4 µm cryosections and identify the ERCs.

### Statistical analysis

All the experimental data were presented as mean ± standard error of the mean (SEM). The results were statistically analyzed by ANOVA test utilizing SPSS version 17.0 software (SPSS Inc., Chicago, USA). *p* < 0.05 was considered statistically significant.

## Results

### ERC treatment improved liver function after ALI

**Fig. 1:**
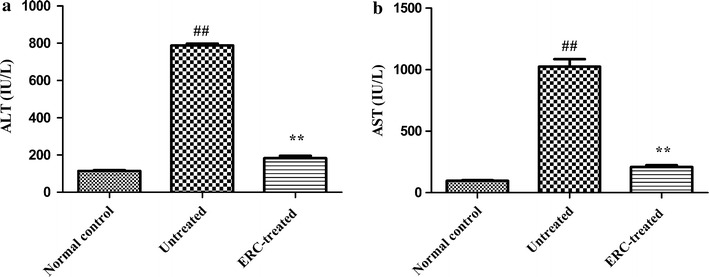
ERCs improved liver function in CCl_4_-induced ALI mice. Serum samples were collected from mice of the normal control, untreated and ERC-treated groups. ERCs significantly reduced serum levels of **a** ALT and **b** AST in comparison with those of untreated ALI group.* Bar graphs* represent mean ± SEM of three separate experiments. *p* values were determined by one-way ANOVA. Data show are representative of three separate experiments performed. (^##^ *p* < 0.01 versus the normal control group. *p < 0.05, **p < 0.01 versus the untreated group, n = 6)

The serum levels of ALT and AST in the untreated group were markedly increased after CCl_4_ injection compared with the normal control group (*p* < 0.01; Fig. 1a, b). In contrast, both serum levels of ALT and AST were significantly decreased by ERC treatment (*p* < 0.01), even though they were still higher than those of normal control group (Fig. 1a, b, *p* < 0.05). In addition, we also found that serum ALT and AST rapidly elevated to peak level 24 h after CCl_4_ treatment, then decreased thereafter, while ERC treatment significantly inhibited the elevation of serum ALT and AST from 24 to 120 h (Data not shown). Taken together, these data suggested that ERCs improve liver function in mice with ALI.

### ERCs ameliorated the histopathological damage of liver tissue after acute injury

**Fig. 2:**
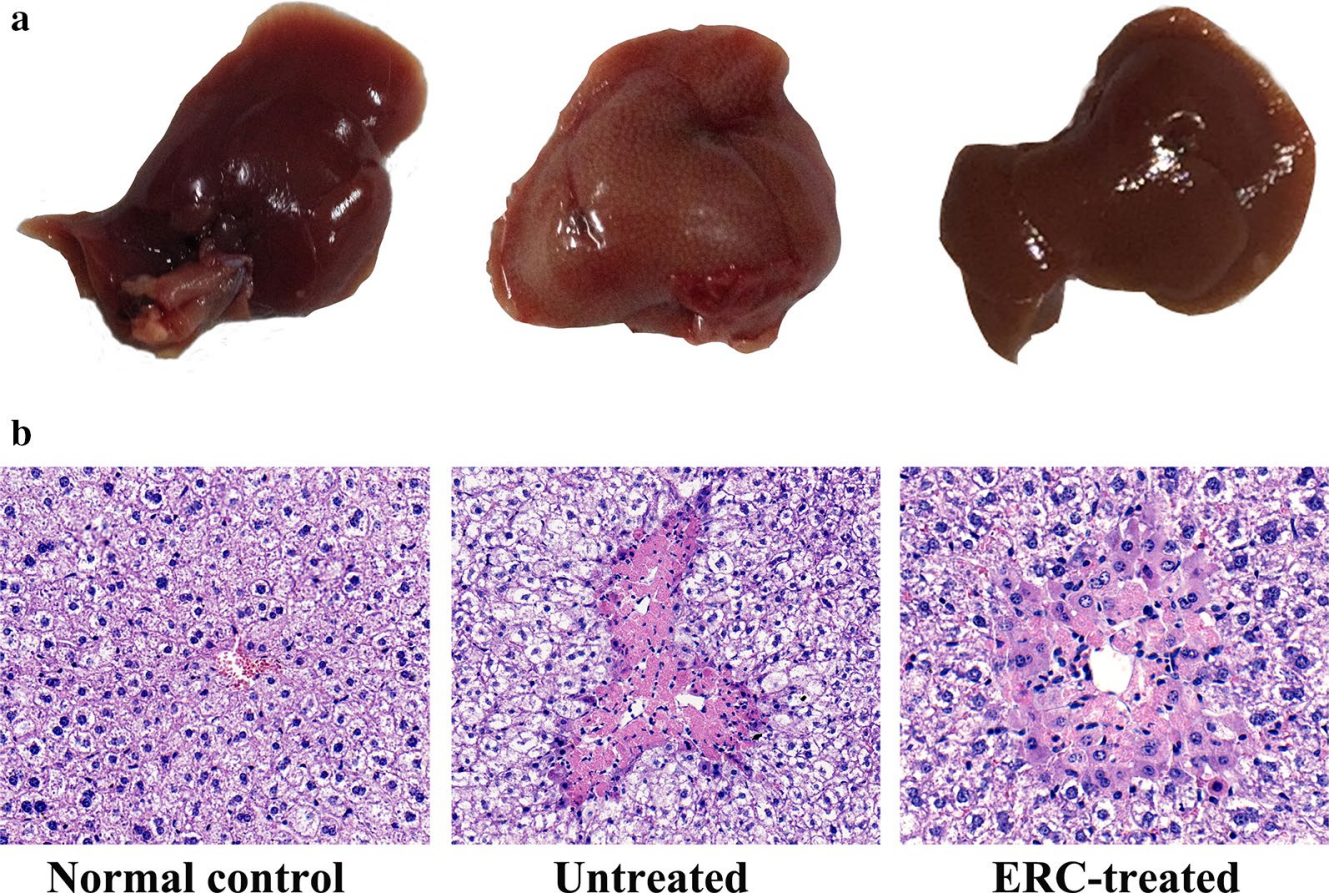
ERC treatment markedly attenuated histopathological damage after CCl_4_ induction. Mice were treated with CCl_4_ (1 ml/kg body weight and 1:3 diluted in corn oil) to induce acute liver injury, then intravenously administered with ERCs (1 × 10^6^/0.2 ml/mouse, suspended in PBS) 30 min after CCl_4_ injection, only once in 24 h. Photographs of livers were taken 24 h after CCl_4_ injection. **a** indicates gross pathological changes of livers. **b** Representative photomicrographs of histological sections of liver (200×, haemotoxylin and eosin staining). Livers in the untreated group exhibited more ballooned hepatocytes, apoptosis and necrosis than those in the normal control groups, which were significantly alleviated by ERC treatment (n = 6)

As shown in Fig. 2, in the untreated ALI group, the liver became inflamed, turned yellowish-white, and increased in volume at 24 h after CCl_4_ injection (Fig. 2a), suggesting that CCl_4_ had induced severe liver cell injury. Notably, the changes observed in the ERC-treated livers were indistinguishable from those in the normal control group (Fig. 2a). In addition, degeneration of liver structure and pathological changes of hepatic parenchymal cells were observed in CCl_4_-induced mice, characterized by hepatocyte necrosis, shrinkage of nuclei, and infiltration of inflammatory cells in the portal area (Fig. 2b). In contrast, all these abnormal changes were alleviated at the same time point after ERC treatment (Fig. 2b). The gross findings and the pathological changes of the liver tissue in ERC-treated group were similar to those of normal controls (Fig. 2a, b). Meanwhile, in consistent with the results of liver function study, we found that the pathological changes of the liver tissue in untreated group at 48, 72, and 120 h time points could be reversed by ERC infusion (Data not shown). Overall, these results indicated that ERC treatment could effectively protect the liver from CCl_4_-induced acute liver damage.

### ERC infusion promoted hepatocyte proliferation in mice with ALI

**Fig. 3:**
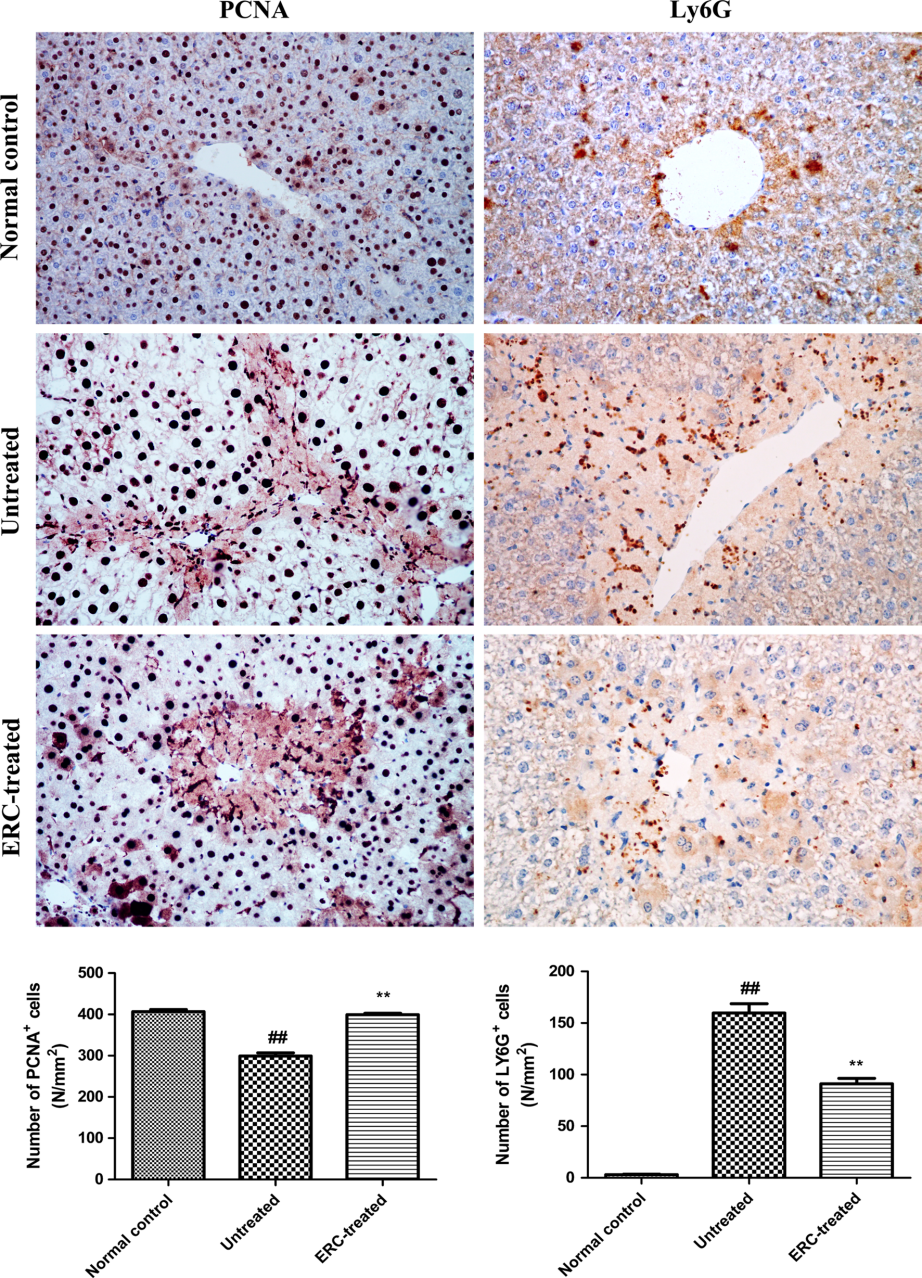
ERCs promoted hepatic cell proliferation and suppressed inflammatory cell infiltration after CCl_4_ injury. Immunohistochemical staining for PCNA and Ly6G were carried out as previous described. PCNA and Ly6G staining of liver sections in the mice with or without ERC administration were performed 24 h after CCl_4_ treatment. The untreated group showed relatively few PCNA^+^ hepatocytes and abundant Ly6G^+^ cells in centrilobular areas. Sections from ERCs treated group exhibited numerous PCNA^+^ hepatocytes surrounding the edge of hepatocellular necrosis and fewer inflammatory cell accumulating in liver tissues. The numbers of PCNA^+^ and Ly6G^+^ cells in the liver sections were measured. At least six 12 mm^2^ tissue sections were counted for each mouse. Values represent mean ± SEM. (^##^ *p* < 0.01 versus the normal control group. *p < 0.05, **p < 0.01 versus the untreated group, n = 6). (Magnification 100×)

To determine whether ERCs have a role in accelerating hepatocyte proliferation after ALI, PCNA expression was detected in the liver tissue by immunohistochemistry. As shown in Fig. 3, compared to the untreated group, ERC infusion dramatically increased the number of PCNA positive-staining cells at 24 h after CCl_4_ induction, with a great number of PCNA positive hepatocytes surrounding the portal area. The changes are indistinguishable from those in the normal control group. This finding demonstrated that treatment with ERCs may markedly promote liver cell proliferation after CCl_4_-induced ALI.

### ERC treatment inhibited neutrophil infiltration in the liver after ALI

To characterize whether ERC treatment could prevent inflammatory cell infiltration following ALI, we performed immunohistochemical staining to evaluate the recruitment of neutrophils in liver tissue. As shown in Fig. 3, ERC treatment notably reduced the number of Ly6G positive cells in the liver compared to those without ERC administration. These results suggested that ERCs significantly reduced neutrophil infiltration in the liver caused by acute liver damage.

### ERC treatment attenuated ALI by regulating cytokine expression

**Fig. 4:**
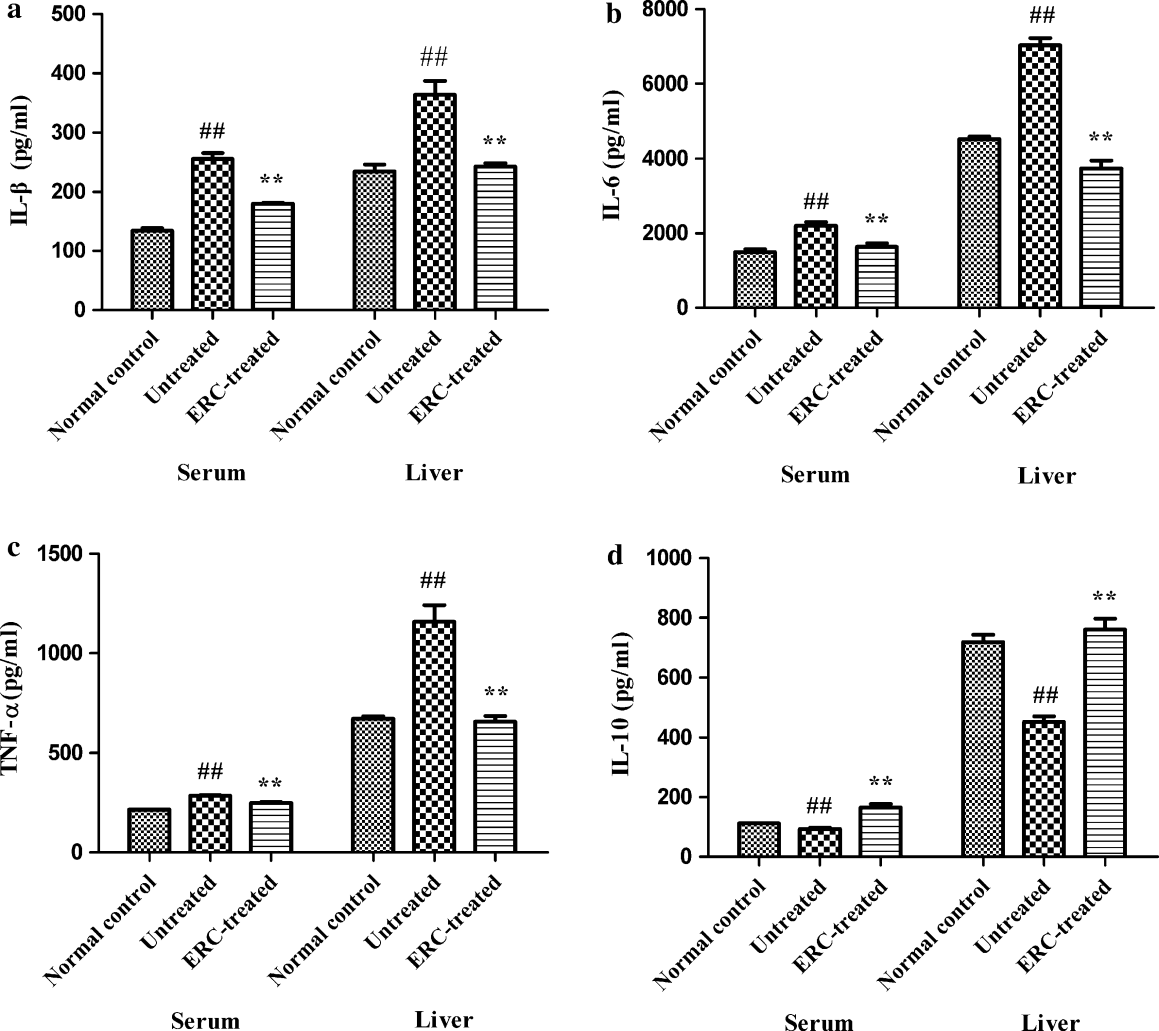
Treatment with ERCs diminished ALI by regulating cytokine profiles. The levels of IL-1β, IL-6, TNF-α,and IL-10 in sera and liver tissues were detected by ELISA at indicated time points after CCl_4_ challenge. Data indicated that ERC treatment significantly decreased the levels of **a** IL-1β, **b** IL-6,and **c** TNF-α, while increased the level of **d** IL-10 in CCl_4_-induced ALI. Values represent mean ± SEM. (^##^ *p* < 0.01 versus the normal control group. *p < 0.05, **p < 0.01 versus the untreated group, n = 6)

To determine whether ERC treatment could affect cytokine profiles, the levels of local and systemic inflammatory cytokines were analyzed and compared among different groups. As shown in Fig. 4, compared with the normal control group, the untreated group exhibited significantly higher levels of pro-inflammatory cytokines (IL-1β, IL-6 and TNF-α) in both the liver (*p* < 0.01) and the serum (*p* < 0.01). In contrast, these cytokine levels were markedly reduced in ERC-treated group (*p* < 0.01, versus untreated group). On the other hand, the level of anti-inflammatory cytokine IL-10 was notably elevated after ERC treatment (*p* < 0.01, versus untreated group). Taken together, these data suggested that treatment with ERCs not only suppress the level of pro-inflammatory cytokines, but also enhance the level of anti-inflammatory cytokine in CCl_4_-induced ALI mice.

### ERC treatment decreased the percentage of CD11c^+^MHCII^+^ cells in the spleen after ALI

**Fig. 5:**
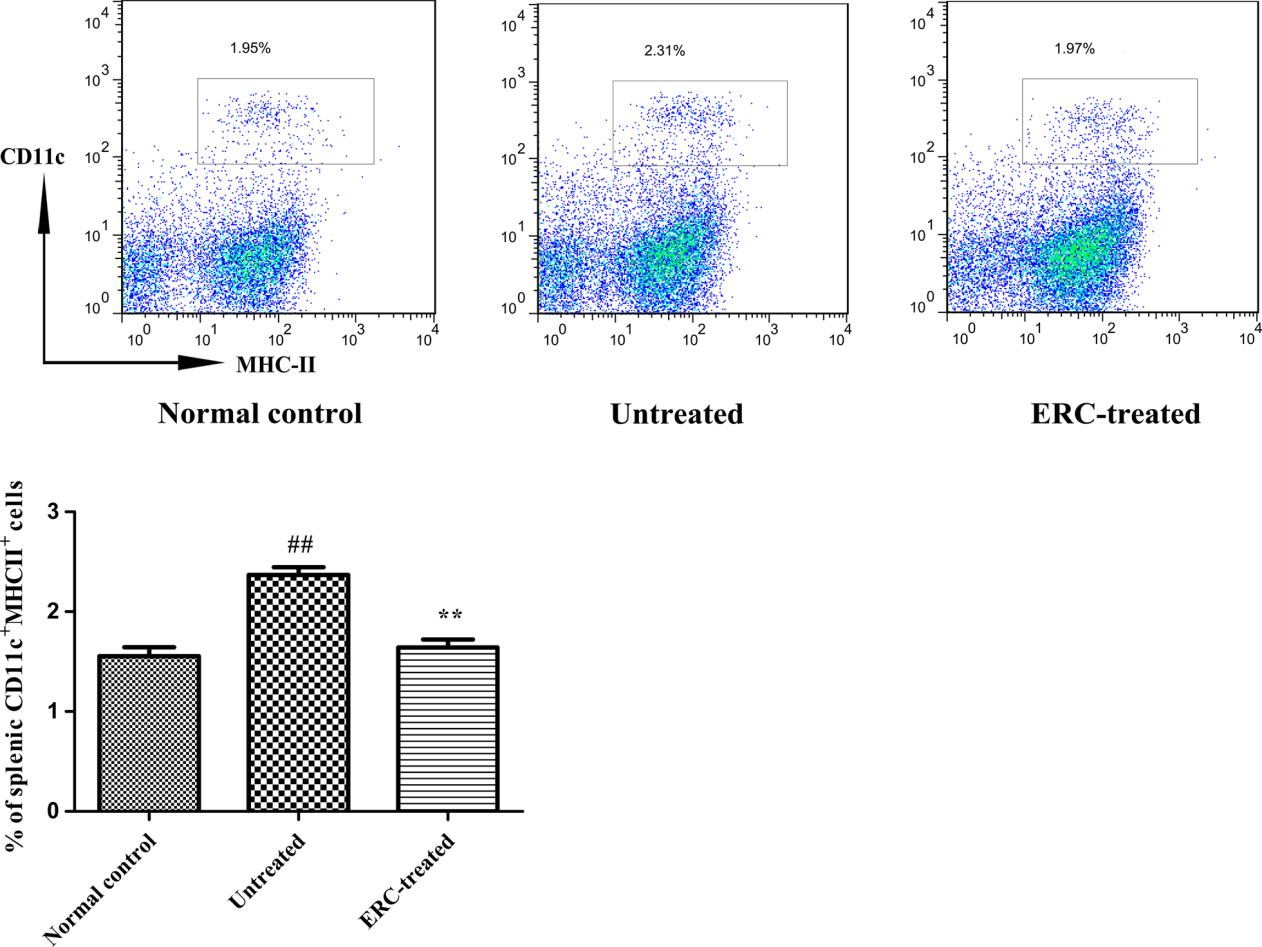
ERCs alleviated ALI by modulating the frequency of DCs. Flow cytometric analysis of splenic DC population was performed in the normal control, untreated and ERC-treated groups. ERC treatment dramaticlly lowered the frequency of CD11c^+^MHCII^+^ cells in ALI mice compared with those of untreated ALI mice.* Bar graphs* represent mean ± SEM of three separate experiments. *p* values were determined by one-way ANOVA. Data show are representative of three separate experiments performed. (^##^ *p* < 0.01 versus the normal control group. *p < 0.05, **p < 0.01 versus the untreated group, n = 6)

Recently Zhang et al. and Nauta et al. demonstrated that MSCs derived from different human tissues reduced the expression of presentation molecules (MHC class II) and co-stimulatory molecules (CD80 and CD86) on mature dendritic cells (DCs) [26, 27]. In this regard, we investigated whether ERC treatment could affect the population of antigen-presenting cells in ALI mice. As shown in Fig. 5, the frequency of MHCII positive DCs in the spleen was significantly lower in ERC-treated mice compared to untreated mice (*p* < 0.01), which suggested that ERC treatment could reduce the population of mature DCs.

### ERCs influenced the populations of CD3^+^CD4^+^ and CD3^+^CD8^+^ T cells in ALI mice

**Fig. 6:**
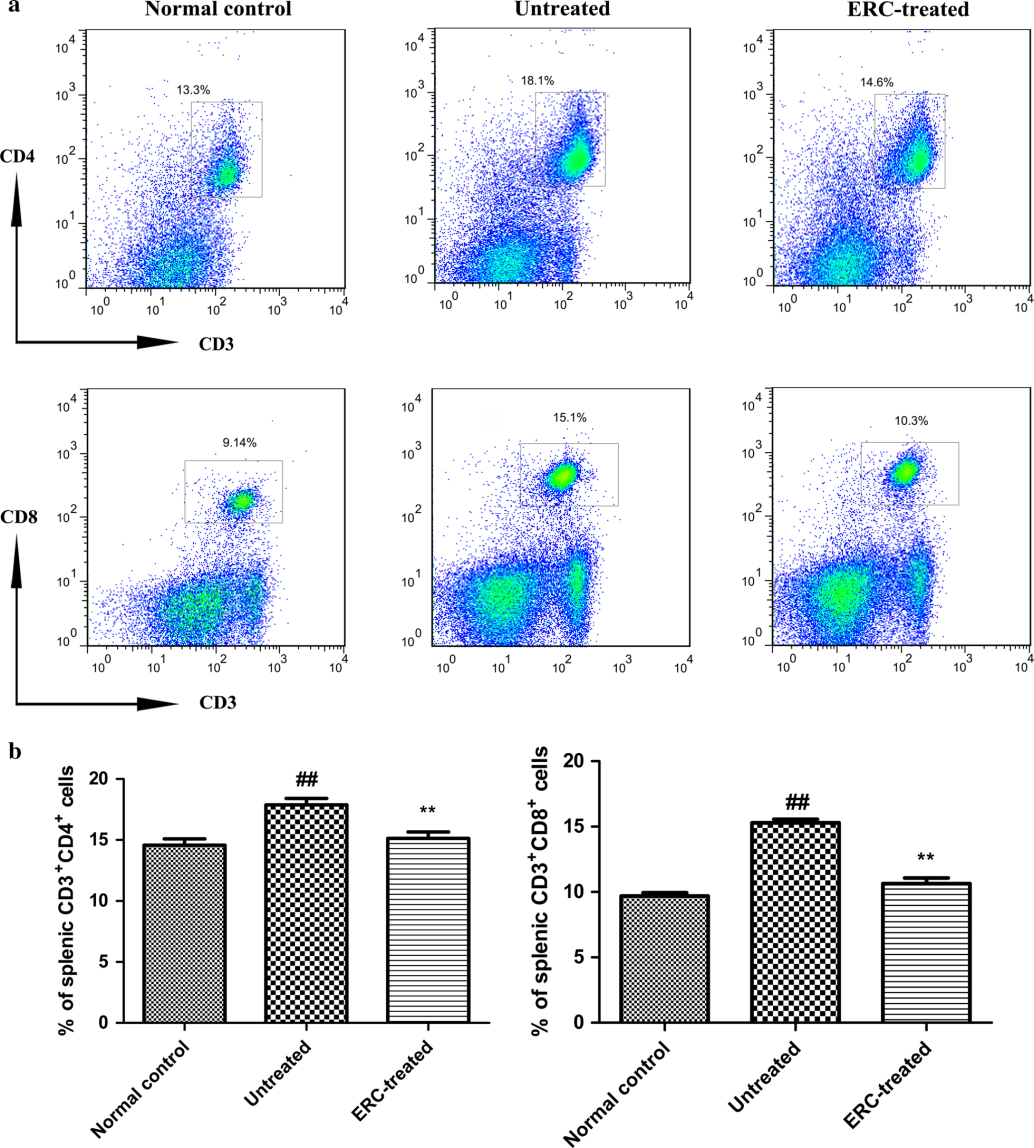
ERCs inhibited the frequency of CD4^+^ and CD8^+^ T cell populations in the spleen. **a** Flow cytometric analysis of CD3^+^CD4^+^ and CD3^+^CD8^+^ T cell populations was performed in the spleen of the normal control, untreated and ERC-treated groups. **b** ERC treatment dramaticlly reduced the percentages of CD3^+^CD4^+^ and CD3^+^CD8^+^ T cells in the spleen.* Bar graphs* represent mean ± SEM of three separate experiments. *p* values were determined by one-way ANOVA. Data show are representative of three separate experiments performed. (^##^ *p* < 0.01 versus the normal control group. *p < 0.05, **p < 0.01 versus the untreated group, n = 6)

To study the relationship between the changes of T cell population and ERC-mediated liver protection, we employed flow cytometric analysis to detect the levels of CD3^+^CD4^+^ and CD3^+^CD8^+^ T cells in both spleen and liver. As indicated in Fig. 6, the percentages of CD3^+^CD4^+^ and CD3^+^CD8^+^ T cells in the spleen were dramatically decreased as compared with those of untreated mice (Fig. 6, *p* < 0.01). However, no difference was observed in the percentages of CD3^+^CD4^+^ and CD3^+^CD8^+^ in the liver in all groups (data not shown).

### ERC treatment upregulated splenic Treg population in ALI mice

**Fig. 7:**
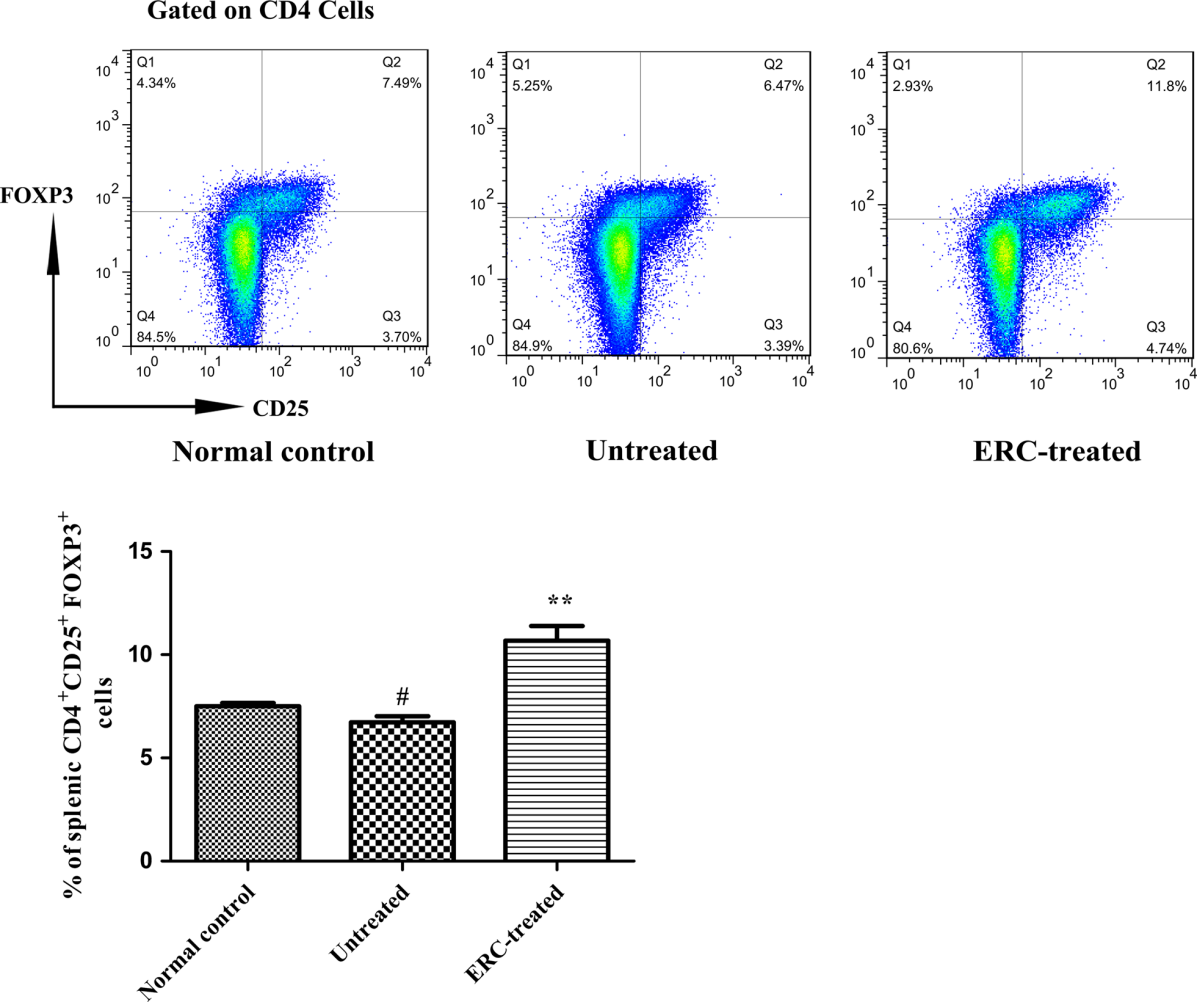
ERCs alleviated ALI by modulating the frequency of Treg population. Flow cytometric analysis of splenic CD4^+^CD25^+^FOXP3^+^Treg population was performed in the normal control, untreated and ERC-treated groups. ERC treatment dramaticlly increase the percentage of Tregs in ALI mice compared with those of untreated ALI mice.* Bar graphs* represent mean ± SEM of three separate experiments. *p* values were determined by one-way ANOVA. Data show are representative of three separate experiments performed. (^##^ *p* < 0.01 versus the normal control group. *p < 0.05, **p < 0.01 versus the untreated group, n = 6)

To further investigate the immunomodulatory function of ERCs in the attenuation of ALI, we measured and compared splenic Treg population among different groups. The percentage of Tregs in the untreated group was much lower than that of the normal control group (Fig. 7, *p* < 0.01). In contrast, the percentage of CD4^+^CD25^+^Foxp3^+^Treg population was significantly increased by ERC treatment in ALI mice (Fig. 7, *p* < 0.01, versus untreated group and normal control group), demonstrating that ERCs have hepato-protective effects in CCl_4_-induced acute liver injury through upregulation of Treg population in mice.

### Tracking in vivo engraftment of ERCs

**Fig. 8:**
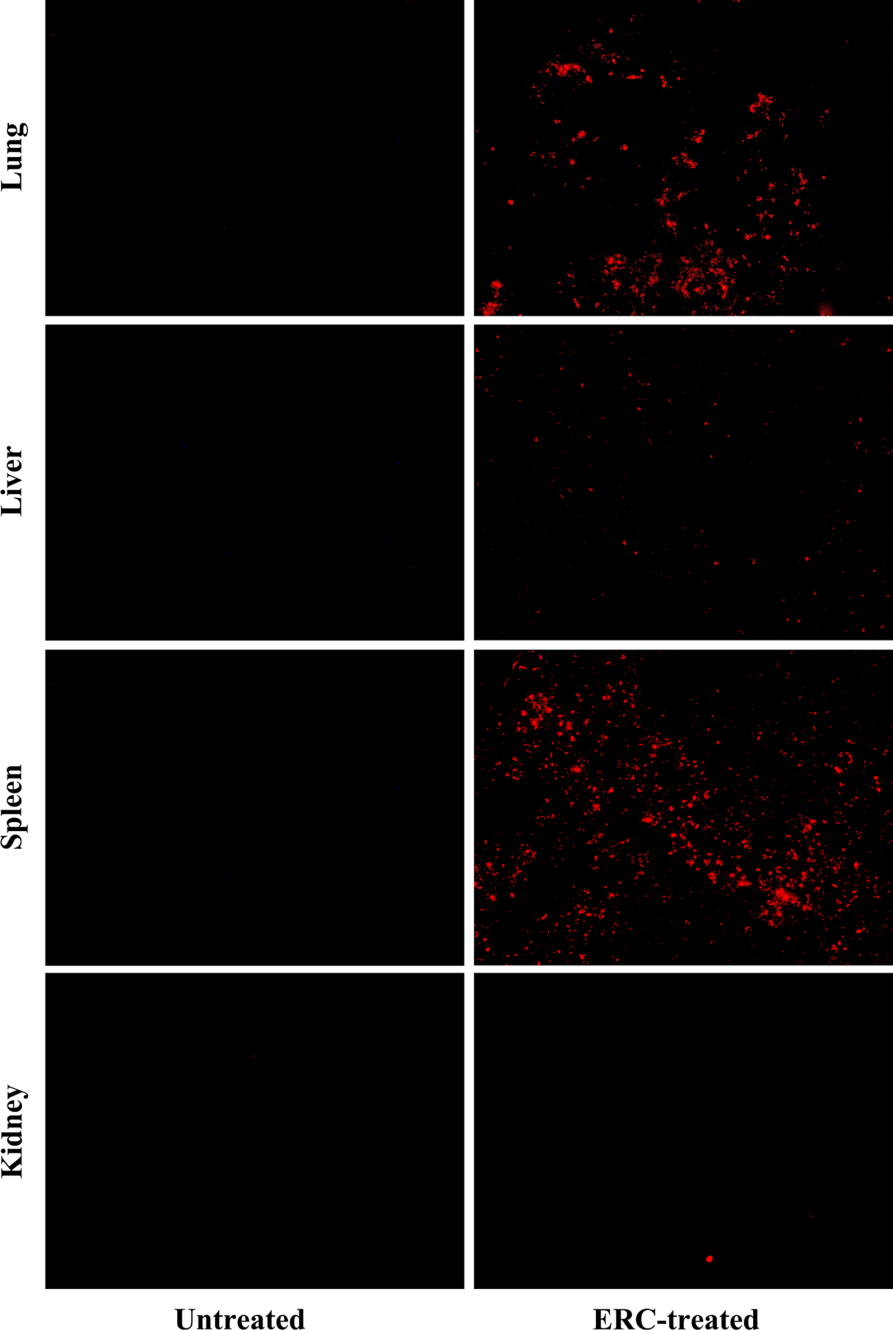
In vivo tracking of PKH26-labeled ERCs in CCl_4_-induced ALI. Frozen section of lung, liver, spleen and kidney from mice of the untreated (as *control*) and ERC-treated groups 24 h after CCl_4_ induction are showed. The strong red flourescent signals indicated that PKH26-positive cells were widely distributed in the hepatic lobules as scattered individual cells 24 h after ALI. Representative image showing PKH26-labeled ERCs were simultaneously observed in murine lung and spleen but not kidney. In addition, no obvious strong red flourescent signals were observed in the lung, liver, spleen or kidney from the untreated group (magnification ×100)

To investigate whether PHK26-ERCs are capable of engrafting CCl_4_-injured liver, animals were sacrificed 24 h after CCl_4_ induction. As shown in Fig. 8, PHK26-positive ERCs were detected by fluorescence microscopy in the liver (injured tissue) and the spleen (lymphoid organ) of ERC-treated mice. Moreover, the labeled ERCs were aslo mainly found in the lung, but not in other normal organs, such as the kidney.

## Discussion

Liver failure can be caused by acute severe or chronic persistent liver injury, while effective treatment are still scarce. Considering the current clinical state, developing an alternative therapeutic strategy to reduce damage, prevent progression, and restore liver function is warranted. Several reports have described the safety and promising beneficial effects of MSCs in the treatment of acute liver injury [6, 28]. However, the value of ERCs, a novel type of MSCs obtained from menstrual blood, in ALI has not been studied. Compared with MSCs from other sources, ERCs have several additional outstanding merits, such as (1) abundant availability, (2) easy and non-invasive acquisition and separation method, (3) higher proliferative rate, (4) relatively unlimited expandability without karyotypic or functional abnormality, (5) more multi-lineage differentiation capacities [29]. In this study, we observed that ERC therapy is an effective strategy for alleviation of ALI. We mainly focused on investigating the therapeutic potential of ERCs related to anti-inflammation, immunomodulation, promotion of hepatocyte proliferation, as well as their engraftment after ERC infusion.

In the present study, we took the advantage of the mouse ALI model to mimic clinical liver dysfunction for evaluating the efficacy of ERC treatment. The mice exposed to CCl_4_ showed significant increase of ALT and AST, which were reduced by ERCs from an early phase of liver injury. Furthermore, livers of the untreated group became inflamed, turned yellowish-white, and increased in volume at 24 h after CCl_4_ injection, suggesting that CCl_4_ had induced severe liver cell injury. Notably, the changes of gross findings observed in ERC-treated livers were indistinguishable from those in the normal control group. In accordance with this finding, the histopathological results demonstrated that ERC administration prominently alleviated cytoplasmic vacuolization, necrosis and infiltration of inflammatory cells. Furthermore, to clarify if the similar beneficial effects of ERC injection be seen longer term, the effects of ERC infusion at different time points have also been studied. The results of biochemical assays and histological examination showed that similar beneficial effects could still be observed 2, 3 and 5 days after ALI induction. Meanwhile, ERCs could still provide a similar benefit when infused 2 h after ALI as 30 min after induction. Taken together, ERCs exhibited liver protective effects on this model of liver damage.

Accumulating evidences indicate that hepatocyte proliferation in stem cell therapy is closely related to increased expression of endogenous and exogenous trophic molecules, including growth factors, transforming growth factor, vascular endothelial growth factor and so on [30]. Similar mechanisms have been reported in acute kidney failure and stroke models [31, 32]. In addition, some in vitro studies also proved that ERCs could differentiate into functional hepatocyte-like cells [13, 33]. To determine the effect of ERCs on liver cell proliferation, we performed PCNA immunohistochemistry. It was found that the population of PCNA positive cells was significantly higher in the ERC-treated group than that of the untreated group, demonstrating that ERCs could promote hepatocyte proliferation.

Neutrophils, a type of phagocytic cell, are potent immune regulators which play an important role in the inflammatory response [34]. Neutrophils have been implicated in several liver injury models such as alcoholic hepatitis [35], ischemia/reperfusion injury of the liver [36], and concanavalin A-induced liver injury [37]. In vivo studies have also exhibited that pathological changes in ALI are significantly improved in neutrophil-depleted mice [36, 38]. Our study demonstrated that ERCs could significantly reduce the numbers of Ly6G-positive cells in the liver compared to that of the untreated group. Therefore, we speculated that ERC treatment contributed to alleviating hepatocellular damage against CCl_4_-induced ALI by suppressing inflammatory cell infiltration.

Local down-regulation of pro-inflammatory cytokines and up-regulation of anti-inflammatory cytokines after MSC transplantation have been described in kidney, lung and liver injury models [31, 39, 40]. To address whether ERCs share the similar attributes in amelioration of liver damage partially through regulating cytokine profiles in the ALI model, we measured the local and serum levels of cytokines. Our data showed that treatment with ERCs dramatically reduced the levels of pro-inflammatory cytokines (IL-6, IL-1β, TNF-α) and increased IL-10, and anti-inflammatory cytokine, compared to those of untreated mice. It has been known that the three acute-phase proteins, IL-1β, IL-6, and TNF-α, are tightly associated with inflammation and cell proliferation and viewed as biomarkers that reflect inflammatory conditions [41]. IL-1β has been previously shown to hamper hepatocyte proliferation [42, 43]. Both IL-1β and IL-1R-deficient mice were not sensitive to inflammatory conditions at the acute phase [44]. IL-6 and TNF-α have also been identified as attractive targets for initiation and progression of liver regeneration. Increasing evidence has shown that IL-6 hyper-stimulation is more likely to cause liver injury [45, 46]. In another study, it was found that ischemia-induced renal damage was ameliorated in IL-6 knockout mice [47]. TNF-α, produced by Kupffer cells (macrophages in liver), acts as a pro-inflammatory mediator in liver apoptosis closely related with cytotoxicity induced by CCl_4_ [48, 49]. Okajima et al. discovered that pretreatment with anti-rat TNF-α antibody could significantly inhibit hepatic I/R [50]. In the current study, the levels of all these three cytokines were reduced by ERC treatment, indicating that ERCs may directly inhibit the pro-inflammatory cytokine secretion to exert liver protective effects.

Meanwhile, previous studies reported that MSCs could secrete IL-10 directly and promote the production of IL-10 by other antigen-presenting cells to exert anti-inflammatory and immunomodulatory effects [51, 52]. It was claimed that IL-10 has a protective function in the liver injury animal model [53]. IL-10 negatively regulates liver regeneration by suppressing production of pro-inflammatory cytokines and inhibiting macrophage and neutrophil recruitment in hepatocytes [54]. The liver protective effect was abolished in IL-10-deficient mice and administration of recombinant IL-10 rescued these mice from chemical-induced hepatitis [25, 51]. In the present study, the levels of IL-10 in the liver and serum were elevated by ERC treatment, suggesting that ERCs may protect the mice from ALI by up-regulating IL-10 both locally and systematically.

Previous studies demonstrated MSCs preferentially integrated into injured liver and enhanced hepatocyte regeneration when infused into CCl_4_ injured mice [28, 55, 56]. Similarly, we transplanted xenogeneic PHK26-ERCs via intravenous injection and found that the transplanted human ERCs quickly migrated into the liver lobules in mice and could be visualized as scattered individual cells 24 h after CCl_4_ administration. Additionally, the level of PCNA positive cells was significantly enhanced after ERC infusion, implying that human ERCs can migrate into the liver and promote liver regeneration in this ALI model. This notion is supported by previous studies that therapeutic effects of ERCs were observed despite utilization of human cells in an immuno-competent xenogeneic animal [13]. Thus, we speculated that ERCs may contribute to hepatocyte proliferation within this damaged environment. In the current study we have also confirmed that ERCs mainly accumulate in the lungs within 24 h after intravenous infusion. This is in accordance with earlier findings that the exogenous fluorescently labelled MSCs remained viable in the lungs up to 24 h after injection [57]. Notably, more fluorescently marked cells were also found in the spleen. Accordingly, the populations of immune cells in spleen were studied to explore the relationship between ERCs and systemic immune reaction.

Dendritic cells (DCs) are the principal antigen-presenting cells in lymphoid organs and periphery including the liver, and are key mediators for the initiation and regulation of both innate and adaptive immune responses [58, 59]. It has been reported that DCs exhibit fibrolytic properties, and the depletion of CD11c^+^ cells in the CCl_4_–induced liver fibrosis model led to slower fibrosis regression and reduced clearance of activated hepatic stellate cells. Conversely, DC expansion induced either by Flt3L (fms-like tyrosine kinase-3 ligand) or adoptive transfer of purified DCs accelerates liver fibrosis regression [60]. In the current study, we evaluated the number of splenic DCs distant from the liver, and observed that the elevation of CD11c^+^MHC-II^+^ DC population after CCl_4_ challenge was significantly reduced by ERC treatment. This is consistent with the finding that MSCs are capable of inhibiting the differentiation of monocytes into DCs [26, 27], suggesting that ERCs probably exert immunomodulatory effects on DCs to control the development of ALI.

T lymphocyte subsets, including CD4^+^ and CD8^+^ T cells, play an important role in the pathogenesis of liver disease [61, 62]. However, the effect of CD4^+^ and CD8^+^ T cells on CCl_4_-induced acute hepatotoxicity in mice remains scarce and even controversial. According to previous studies, antigen-specific CD8^+^ T cells migrate to the contact site upon re-exposure to the chemicals and cause tissue damage through the release of cytokines and cytolytic molecules [63–66]. Researchers used an anti-CD8 monoclonal antibody to neutralized CD8 T cells and demonstrated that depletion of CD8 T cells protected mice from Amodiaquine-induced liver injury [67]. Results from other experiments confirmed that CD4^+^ T cell depletion was capable of ameliorating the extent of injury with less neutrophil infiltration after I/R liver damage; however, liver damage was reproduced when adoptive transfer of CD4^+^ lymphocytes to CD4 knockout mice [68, 69]. In our study, as compared with the untreated ALI group, ERC treatment group experienced a significant reduction in CD4^+^ and CD8^+^ T cells, indicating that ERCs may inhibit T cell accumulation. The findings suggested that ERCs may have regulatory functions on the cell populations of splenic CD4^+^ and CD8^+^ T cells. Similar results were also found in animal models with renal I/R injury and ulcerative colitis [15, 20]. Meanwhile, this study also proved that, like MSCs, ERCs possess immunomodulatory properties which could suppress the activation and proliferation of T cells [70].

Tregs are believed to play a critical role in the suppression of both innate and adaptive immune responses [71], and are also an important factor in the attenuation of liver injury [72–74]. CD4^+^CD25^+^ Tregs account for 5–10 % of the CD4^+^ T cell panel in healthy humans and mice, which is sufficient to maintain immune homeostasis and limit autoimmune disease [75]. The role of Tregs in ALI has been confirmed in several studies using PC61, an anti-CD25 monoclonal antibody that depletes Tregs before liver damage, to verify the protective effect of Tregs. It was found that mice suffering from Treg depletion experienced an aggravation of ALI compared to ALI mice that did not have Treg depletion [25]. In another study, the protective effects of Tregs on ALI were confirmed via the adoptive transfer method [76]. Similarly, our results demonstrated that CD4^+^CD25^+^Foxp3^+^ Tregs were significantly decreased in the untreated-ALI mice compared to the normal control mice, and significantly elevated in ERC-treated mice, indicating that ERC treatment mitigated CCl_4_-induced acute hepatotoxicity in mice by increasing the population of Tregs. Overall, we speculate that transplanted PKH26-labeled ERCs engraft to the spleen in mice with ALI and interact with immune cells, leading to the downregulation of splenic CD11c^+^MHC-II^+^ DCs, CD4^+^ and CD8^+^ T cell population, as well as the upregulattion of Treg population. In the meantime, since ERC supernatant could still exert similar beneficial effects on ALI as compared to the effects achieved by cell infusion (Data not shown), ERC treatment may also attenuate ALI by releasing immunomodulatory cytokines. Experiments to better understand the mechanisms of ERC-mediated immunomodulation in this ALI model are underway.

## Conclusions

In conclusion, our study demonstrated that human ERCs are effective in treating CCl_4_-induced ALI. ERCs improved liver function and attenuated pathological changes by promoting liver cell regeneration, modulating cytokine profiles, and regulating immune cells. However, further studies are still needed to elucidate the complex pathways underlying ERC-mediated liver protective effects at molecular levels. Taken together, these findings may provide a rationale for the use of ERCs in clinical settings.

## Abbreviations

ALI: acute liver injury
ALT: alanine aminotransferase
AST: aspartate aminotransferase
CCl_4_: carbon tetrachloride
DCs: dendritic cells
ERC: endometrial regenerative cells
IL: interleukin
Ly6G: lymphocyte antigen 6 complex, locus G
MSCs: mesenchymal stem cells
PCNA: proliferating cell nuclear antigen
Treg: regulatory T cell
TNF: tumor necrosis factor
